# COVID-19 Vaccinating Russian Medical Students—Challenges and Solutions: A Cross-Sectional Study

**DOI:** 10.3390/ijerph191811556

**Published:** 2022-09-14

**Authors:** Olesya V. Kytko, Yuriy L. Vasil’ev, Sergey S. Dydykin, Ekaterina Yu Diachkova, Maria V. Sankova, Tatiana M. Litvinova, Beatrice A. Volel, Kirill A. Zhandarov, Andrey A. Grishin, Vladislav V. Tatarkin, Dmitriy E. Suetenkov, Alexander I. Nikolaev, Michael Yu Pastbin, Innokenty D. Ushnitsky, Svetlana N. Gromova, Gulshat T. Saleeva, Liaisan Saleeva, Nail Saleev, Eduard Shakirov, Rinat A. Saleev

**Affiliations:** 1 Sklifosovskyi Institute of Clinical Medicine, I.M. Sechenov First Moscow State Medical University, St. Trubetskaya, 8, bld. 2, 119991 Moscow, Russia; 2 Department of Operative and Clinical Surgery with Topographic Anatomy Named after S.A. Simbirtsev, Mechnikov North-West State Medical University, Kirochnaya St., 41, 191015 Saint-Petersburg, Russia; 3 Department of Pediatric Dentistry and Orthodontics, V.I. Razumovsky Saratov State Medical University, B. Kazachya St., 112, 410012 Saratov, Russia; 4 Department of Therapeutic Dentistry, Smolensk State Medical University, Krupskoy St., 28, 214019 Smolensk, Russia; 5 Department of Children Dentistry, Northern State Medical University, Troitsky Avenue, 51, 163000 Arkhangelsk, Russia; 6 Department of Therapeutic, Surgical and Prosthetic Dentistry, M.K. Ammosov North-Eastern Federal University, Belinsky St., 58, 677000 Yakutsk, Russia; 7 Department of Dentistry, Kirov State Medical University, K. Marx St., d.112, 610998 Kirov, Russia; 8 Department of Prosthetic Dentistry, Kazan State Medical University, Butlerova St., 49, 420012 Kazan, Russia

**Keywords:** COVID-19 pandemic, COVID-19 vaccination coverage, COVID-19 vaccination problems, epidemiological indicators, medical students, SARS-CoV-2 immunoprophylaxis

## Abstract

*Background*: The role of preventive measures increases significantly in the absence of effective specific COVID-19 treatment. Mass population immunization and the achievement of collective immunity are of particular importance. The future development of public attitudes towards SARS-CoV-2 immunization depends significantly on medical students, as future physicians. Therefore, it seemed relevant to determine the percentage of COVID-19-vaccinated medical students and to identify the factors significantly affecting this indicator. *Methods*: A total of 2890 medical students from years one to six, studying at nine leading Russian medical universities, participated in an anonymous sociological survey. The study was performed in accordance with the STROBE guidelines. *Results:* It was found that the percentage of vaccinated Russian medical students at the beginning of the academic year 2021 was 58.8 ± 7.69%, which did not significantly differ from the vaccination coverage of the general population in the corresponding regions (54.19 ± 4.83%). Student vaccination rate was largely determined by the region-specific epidemiological situation. The level of student vaccination coverage did not depend on the gender or student residence (in a family or in a university dormitory). The group of senior students had a higher number of COVID-19 vaccine completers than the group of junior students. The lack of reliable information about COVID-19 vaccines had a pronounced negative impact on the SARS-CoV-2 immunization process. Significant information sources influencing student attitudes toward vaccination included medical professionals, medical universities, academic conferences, and manuscripts, which at that time provided the least information. *Conclusion:* The obtained results make it possible to develop recommendations to promote SARS-CoV-2 immunoprophylaxis among students and the general population and to increase collective immunity.

## 1. Introduction

The long-term pandemic of the new coronavirus infection caused by the single-stranded RNA-containing virus SARS-CoV-2 has become a serious challenge not only for the healthcare systems but also for the economies of all countries [1]. Unprecedented measures are being taken to organize medical care for SARS-CoV-2-infected people and to rehabilitate patients with severe post-COVID complications [2]. In the absence of effective specific COVID-19 treatment, the role of prophylaxis increases significantly. Along with nonspecific prevention methods and compliance with sanitary–epidemiological requirements, mass population immunization and the achievement of collective immunity are of particular importance [3,4].

Over the years of the pandemic, a significant amount of scientific information has been accumulated on the SARS-CoV-2 pathogenesis, the virus biology, and its interaction with the human immune system, making it possible to create effective COVID-19 vaccines [5,6,7]. In Russia, the first COVID-19 vaccine was officially recognized in August 2020. It was the two-component “Gam-COVID-Vac” (“Sputnik V”), based on the safe rAD26-S и rAD5-S adenovirus carriers and developed by the N.F. Gamaleya Federal Research Center for Epidemiology & Microbiology. The main advantage of such viral vector vaccines is their natural mechanism of transporting SARS-CoV-2 genetic fragments into human cells, allowing for sufficient long-term antigen expression, effectively activating innate and adaptive immunity [8,9,10]. Then, in October 2020, the genetically engineered protein vaccine “EpiVacCorona”, made from three different artificial peptides copying SARS-CoV-2 fragments, was registered by the by State Research Center of Virology and Biotechnology “Vector”. Persons over 18 years of age without contraindications are allowed to be vaccinated with the “Sputnik V” and “EpiVacCorona” vaccines [11,12]. The most traditional technological platform for creating vaccines was used by the Chumakov Federal Scientific Center for Research and Development of Immune and Biological Products of the Russian Academy of Sciences, which developed an inactivated whole-virion vaccine, “CoviVak”, that became available in February 2021 for persons from 18 to 60 [13,14,15,16]. All these vaccines are administered twice intramuscularly at 2–3 weeks’ interval [8,10,12,17,18,19]. In May 2021 the first component of the “Sputnik V” vaccine was registered as “Sputnik Light”, which was intended for revaccination or vaccination of young people (18–30 years old), whose immunity is well formed, and one injection is enough. Later, “EpiVacCorona” was optimized into “EpiVacCorona H”, in which two of the three peptides were combined into one [9,10,19,20,21,22,23,24,25]. In Moscow, COVID-19 vaccination began on 5 December 2020; in other Russian regions, this was on 10 December, according to the unified COVID-19 immunization program. Military, teachers, health care workers, and social workers were the first in the vaccination campaign. SARS-CoV-2 immunization became available to everyone in Russia in January 2021. Persons who have completed the full COVID-19 vaccination course receive a special certificate that is required in order to be able to visit public places and educational institutions [9,10,11,13,14,15].

A special risk group in the pandemic situation is medical students who have a high infection risk due to their frequent visits to different patients and emergency practice in coronavirus hospitals [26,27,28]. Medical students, as future physicians, are essential in forming public attitudes towards SARS-CoV-2 immunization since there is currently a worldwide problem of insufficient vaccination coverage due to mistrust and deliberate avoidance of this highly effective measure [29]. In this regard, it was particularly important to study the real COVID-19 vaccination rates among medical students during the SARS-CoV-2 pandemic. The goal of this study was to determine the percentage of COVID-19-vaccinated medical students and to identify the factors that significantly affect this indicator in order to be able to develop recommendations that will help to increase vaccination rates among the population and achieve the target of collective immunity.

## 2. Materials and Methods

### 2.1. Study Design and Participants

**Figure 1:**
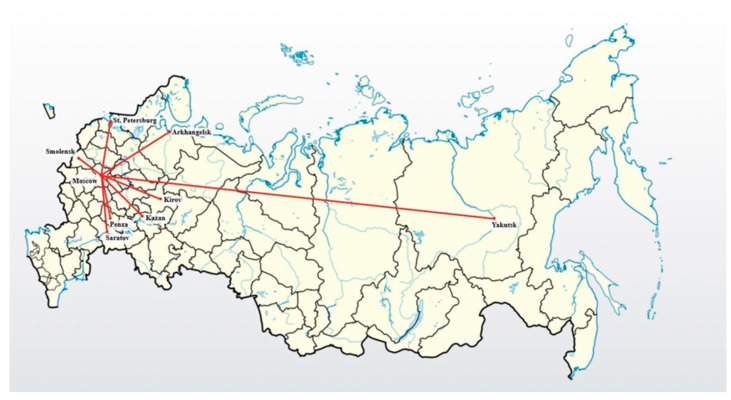
Regions of online medical student survey in Russia.

A total of 2890 1st- to 6th-year medical students from nine leading Russian universities located in the regions with different epidemiological SARS-CoV-2 situation were included in a cross-sectional online anonymous survey conducted in the period of late September−early October 2021 (Figure 1). By this time, students had had the opportunity to be vaccinated against COVID-19 for more than six months from the start of vaccination.

**Figure 2:**
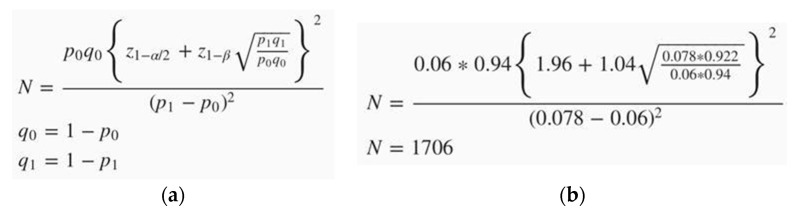
Sample size count: (**a**) ‘classic’ formula for counting; (**b**) formula and counting process for present study.

The study was carried out on the Google Forms platform (Alphabet, Mountain View, California, USA) in accordance with STROBE guidelines. The reference to the questionnaire was distributed among students through social networks and Internet information channels. Using G*Power software statistical package (ChristianAlbrechts-Universität, Olshausenstr, Germany) [30] and based on a moderate effect size 0.3, power 85%, and alpha < 0.05, the minimum sample size needed for this study was calculated to equal 1706 student that was approximately 7.52% of the total number of students (22,694 students) enrolled in the nine institutes. The formula used and the calculations for the minimum sample size are shown below (Figure 2).

Respondent sample representativeness in each of the nine universities is presented in Table 1.

### 2.2. Epidemiological Situation Characteristics in Studied Russian Regions

The up-to-date data provided by Russian Federal Service on Customers’ Rights Protection and Human Well-Being Surveillance (Russian Federal Service on Customers’ Rights Protection and Human Well-Being Surveillance—the up-to-date data are available at: https://xn--80aesfpebagmfblc0a.xn--p1ai/information/, accessed on 15 November 2021) were used to identify the correlation between student behavioral attitudes toward SARS-CoV-2 immunization in relation to morbidity, mortality, lethality, and vaccination population coverage of the corresponding Russian region.

**Figure 3:**
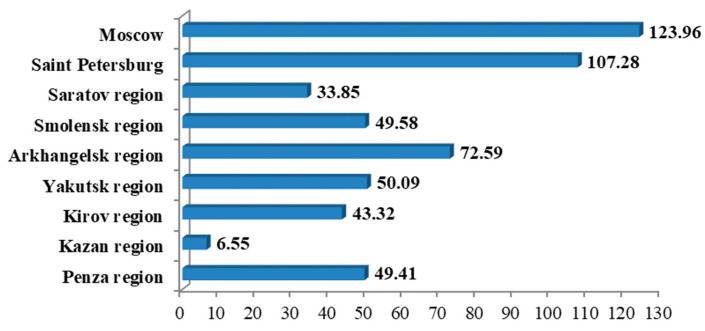
Total SARS-CoV-2 incidence per 1000 people at the time of the study.

**Figure 4:**
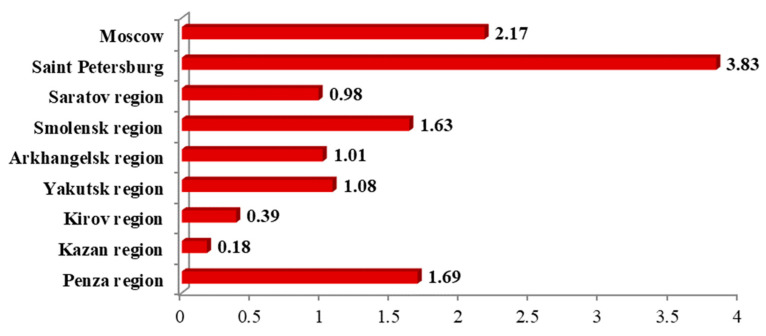
Total mortality because of SARS-CoV-2 per 1000 people at the time of the study.

Moscow and Saint Petersburg led steadily with respect to numbers of patients and number of deaths per 1000 people among the Russian regions studied (*p* < 0.05). Minimum incidence and mortality were observed in the Tatarstan Republic (*p* < 0.05), where Kazan State Medical University is located (Figure 3 and Figure 4).

**Figure 5:**
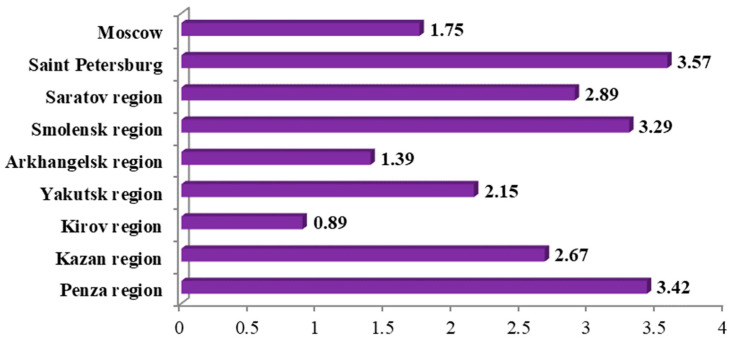
Lethality because of SARS-CoV-2 at the time of the study.

The highest lethality rates, defined as the ratio of deaths occurring because of SARS-CoV-2 to the total number of people affected by the disease during the pandemic period, were recorded in Saint Petersburg, Penza, and Smolensk regions (*p* < 0.05). The minimum value of this indicator was observed in the Kirov region (*p* < 0.05) (Figure 5).

**Figure 6:**
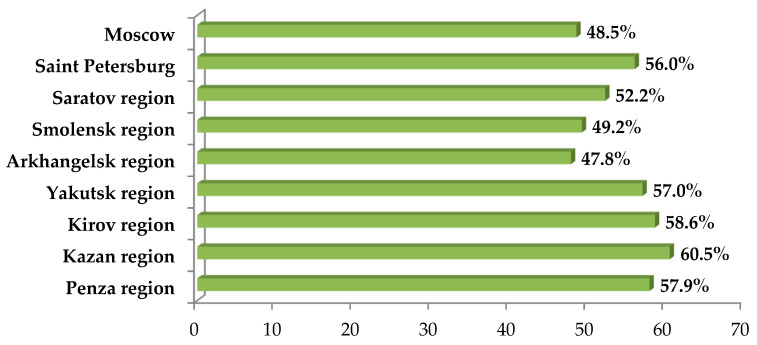
Vaccination coverage of the populations of the studied regions at the time of the study.

COVID-19 vaccination coverage of the population of each region at the time of the study is shown in Figure 6. It should be emphasized that the proportion of vaccinated people in Moscow, Smolensk and Arkhangelsk regions was significantly lower than in the Tatarstan Republic, Kirov and Penza regions. On average, the percentage of citizens that had been vaccinated in the studied regions was 54.1%, while in the whole of Russia this figure was 52.3%.

### 2.3. Questionnaire Development and Content

The original questionnaire was designed using prior scientific studies on vaccine and behavioral attitudes toward immunization, with new factors added [26,27,28,31,32,33,34,35]. Personal information such as institutional affiliation, academic year, student residence, age, and sex were requested to be filled in first (Supplementary Materials). The main part of the questionnaire consisted of four sections, the first of which contained questions about COVID-19 assessment, previous COVID-19 experience in relatives and themselves, attitudes towards non-specific SARS-CoV-2 preventive measures, and the evaluation of their effectiveness. In the second section, student beliefs about COVID-19 vaccination were studied. To identify their determination of the effectiveness of vaccination and non-specific SARS-CoV-2 preventive measures, students used a visual analog scale, in which 10 points corresponded to the effectiveness maximum, and 0 points to its complete absence. The third section focuses on participant COVID-19 vaccine preference and vaccine information sources. Finally, we investigated COVID-19 vaccination coverage among medical students, side effects after COVID-19 vaccination, and predominant reasons for not vaccinating. In the final questions, respondents were able to choose several options. To ensure questionnaire content validity, a pilot study was carried out on 36 students who were not included in the final study. All questions were checked for clarity and ease of understanding by three independent experienced experts.

### 2.4. Ethical Considerations

The work fully complied with the requirements of the local ethical committee of Sechenov University (Protocol No. 04-19 dated 6 March 2019) and the norms of the Declaration of Helsinki. All students were recruited on a volunteer basis and gave informed consent before the study. Respondents understood the survey purpose and were told how to fill in the questionnaire. No reward was offered to participants.

### 2.5. Statistical Analysis

Statistical data analysis was performed using SPSS 20.0 (SPSS Inc., Chicago, IL, USA) and MedCalc 11.5.00 (MedCalc Software, Oostende, Belgium). The minimal subject number needed for this study was calculated by means of power analysis. Descriptive statistics had a place for data from scales where the mean, median, standard deviation, and percentiles were calculated. Intergroup qualitative indicators were compared using Pearson’s χ-squared test or Fisher’s exact test. The normality of distributions was checked with Kolmogorov–Smirnov test. Quantitative differences were determined using the independent t-test for normal distribution or the non-parametric Mann–Whitney U-test when variables were non-normally distributed. For multiple-comparison, non-parametric Kruskal–Wallis test and parametric ANOVA were used. Pearson’s coefficient and Spearman’s rank test were calculated for correlation analysis. Statistics were considered significant at *p* < 0.05. The results were counted twice by 2 independent researchers. The inter- and intragroup correlation agreement rates were greater than 95%; for this reason, all results were considered as the mean between 2 attempts by 2 researchers.

## 3. Results

### 3.1. Participant Characteristics

The mean respondent age was 20.99 ± 2.28 years (range of 16 to 37 years) (Table 2). The survey sample included students from the 1st to 6th year; therefore, two subgroups of junior (1–3 year—1467 (50.8%)) and senior (4–6 year—1423 (49.2%)) students were formed to determine the dependence of commitment to COVID-19 vaccination on the professional training level. There were 800 males (27.7%) and 2090 females (72.3%), which was used to examine the proportion vaccinated according to sex.

### 3.2. Participant COVID-19 Experience and Non-Specific SARS-CoV-2 Prophylaxis Evaluation

Most students consider COVID-19 a dangerous disease (89.4%) and express profound concern about the current unfavorable sanitary–epidemiological situation (89.9%) associated with the rapid spread of new coronavirus variants. Three-quarters of the respondents have already encountered this disease in their relatives (75.9%), and every third had experienced the death of a loved one (36.98 ± 5.79%). Almost half of the young surveyed people (48.6%) had experienced RT-PCR test-confirmed COVID-19 themselves, with one in two (46.6%) being diagnosed with moderate and severe form of this disease (according to computed tomography criteria). Most students (85.4%) are responsible with respect to following non-specific SARS-CoV-2 preventive measures, which primarily include wearing masks in public places, treating hands with antiseptic, and social distancing. Respondents rated the effectiveness of these anti-COVID measures at 4.73 ± 0.51 points (the median was 5.0 (IQR, 3.0–7.0).

### 3.3. Participant Beliefs about COVID-19 Vaccination

Most future specialists (65.0%) consider vaccination to be the most effective method for infectious disease prevention; every second respondent (57.3%) sees the immunization expediency even directly during a pandemic. However, only a little more than a third of all students (39.0%) expressed a definite positive attitude towards COVID-19 vaccination, and the same number of respondents (42.7%) have not yet fully decided on this issue, so only half of all respondents (52.5%) recommend their relatives, friends, and acquaintances to vaccinate against coronavirus infection.

The effectiveness of SARS-CoV-2 specific prophylaxis was estimated by medical students at 4.87 ± 0.50 points (the median was 5.0 (IQR, 3.0–7.0), which did not differ significantly from the effectiveness of non-specific SARS-CoV-2 preventive measures (4.73 ± 0.51, respectively; *p* > 0.05). Many future doctors (66.5%) believe that COVID-19 vaccine prophylaxis acts as a necessary guarantee of protection against moderate and severe disease forms, fatal outcomes, and post-infectious complications. Only a tenth of the respondents (10.9%) suppose that active immunization can prevent disease incidence.

**Figure 7:**
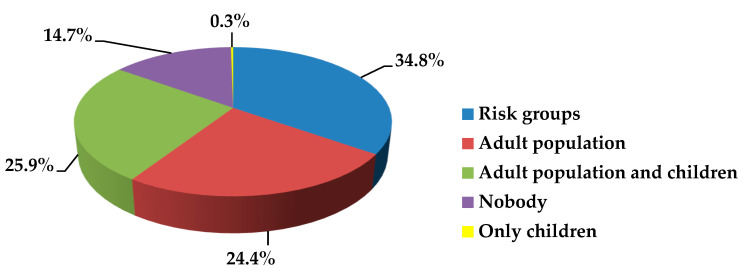
Population groups to be vaccinated against COVID-19.

Just over half of the students (59.2%) indicate that the COVID-19 vaccination need is primarily for at-risk individuals and adults. A quarter of respondents note that both adults and children should be vaccinated. One in six students (14.7%) is convinced that SARS-CoV-2 immunization is not indicated for anyone at all (Figure 7). Almost all students (86.5%) emphasize that vaccination should be voluntary only.

### 3.4. Participant COVID-19 Vaccine Preference and Vaccine Information Sources

The highest percentage of (41.9%) medical students express the opinion that all COVID-19 vaccines are effective regardless of the origin country, 18.3% respondents are convinced of the good quality of only Russian vaccines, 24.3% respondents trust only imported vaccines. Every sixth medical student (15.5%) believes that all immunobiological agents are ineffective.

**Figure 8:**
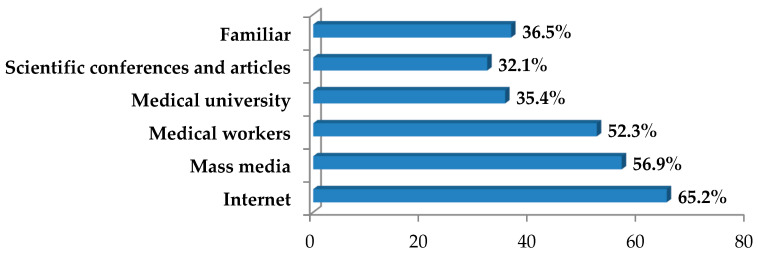
Key information sources of COVID-19 vaccines in medical students.

Every third medical school student (31.9%) reported a clear lack of reliable information about anti-COVID vaccines, their composition, action mechanisms and contraindications. Sources of COVID-19 preventive vaccines, used by medical students for subsequent informed consent or refusal to participate in a vaccination campaign against COVID-19, were identified (Figure 8).

Thus, medical students received most information about active SARS-CoV-2 immunization drugs from the Internet and the mass media. Half of the respondents, in order to make their choice (52.3%), relied on information obtained from medical professionals. The least importance in obtaining knowledge about vaccines was found for medical universities, scientific conferences and manuscripts, the role of which is reported only by a third of students, which is comparable to the number of students whose source of information is familiar. Half of future doctors (47.7%) are not satisfied with the quality of information they received at all.

### 3.5. COVID-19 Vaccination among Medical Students and Reasons for Not Vaccinating

**Figure 9:**
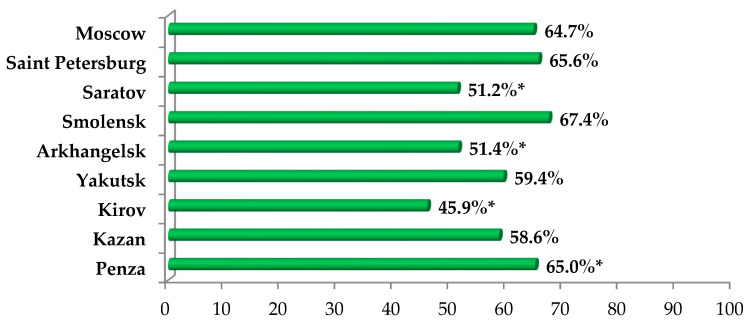
The distribution of vaccinated medical students by Russian region. Note: *—the differences are significant, *p* < 0.05.

The COVID-19 vaccination coverage among Russian medical students was 58.8%, which did not significantly differ from the percentage of citizens who were vaccinated in the studied regions, which was 54.2% (*p* < 0.05). The distribution of the vaccinated by the region in Russia is shown in Figure 9. It should be noted that the percentage of preventive COVID-19 vaccination among the students of Saratov, Kirov and Northern State Medical University is significantly lower than that at other universities (*p* < 0.05).

The percentage of COVID-19-vaccinated students was not dependent on gender (57.9% and 61.3%, respectively; *p* > 0.05), and was significantly higher in the group of senior students compared to junior students (68.7% and 49.2%, respectively; *p* < 0.05). It was found that in a significantly larger percentage of cases, students were motivated to receive the COVID-19 vaccination certificate than to prevent the disease and its severe course (59.2% and 45.4%, respectively; *p* < 0.05).

**Figure 10:**
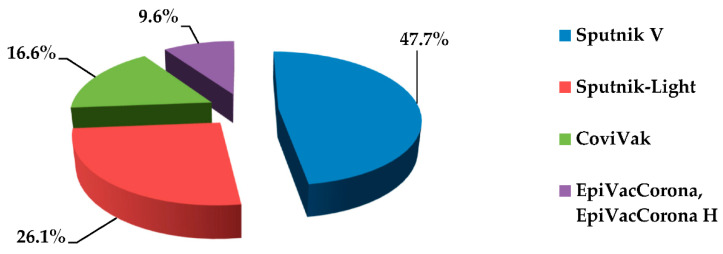
COVID-19 vaccines selected by medical students.

The most popular vaccines chosen by medical students for the SARS-CoV-2 prevention were the combined two-component vaccine “Gam-COVID-Vac” (“Sputnik V”) and the single-component vaccine Sputnik-Light, based on viral vectors and developed by the N.F. Gamaleya Federal Research Center for Epidemiology & Microbiology (Figure 10). In second place was the medication “CoviVak”, an inactivated whole-virion vaccine, registered by the Chumakov Federal Scientific Center for Research and Development of Immune and Biological Products of Russian Academy of Sciences. The subunit (protein) vaccines “EpiVacCorona” and “EpiVacCorona H”, developed by the by State Research Center of Virology and Biotechnology “Vector”, were the least in demand.

The most common side effects after COVID-19 vaccination were weakness (71.4%), injection site soreness (66.2%), fever (64.5%), headache (54.1%) and muscle aches (52.2%), which, as a rule, disappeared on their own within 2–3 days. In 14.8% of respondents, side effects were completely absent.

The predominant reasons for not vaccinating were lack of awareness about COVID-19 vaccines and doubts about their efficacy and safety (15.3%), fear of side effects and post-vaccination complications (23.5%), presence of post-COVID immunity (15.3%) and medical withdrawal from vaccination due to existing contraindications (12.0%).

### 3.6. Identifying Factors Affecting COVID-19 Vaccination Coverage among Medical Students

Multicomponent correlation analysis made it possible to identify the factors affecting the COVID-19 vaccination coverage among Russian medical students. Thus, it was shown that the student attitude towards COVID-19 vaccination was almost independent of their opinion about the danger of this coronavirus infection (r = −0.009) and their concern about the current sanitary–epidemiological situation (r = −0.235). It was found that students were motivated to carry out specific prophylaxis not so much by an increase in the overall incidence in the region (r = 0.388) as by the incidence among students themselves (r = 0.573) and their relatives (r = 0.626). Increased mortality rates because of SARS-CoV-2 (r = 0.655) and, especially, the percentage of deaths among close people (r = 0.778) had a great effect. It should be noted that there is a strong positive correlation between the percentage of vaccinated medical students and the lethality rate because of SARS-CoV-2 in this region (r = 0.701). There was no relationship between the SARS-CoV-2 immunization coverage of the region population and the proportion of SARS-CoV-2 vaccinated medical students (r = −0.119).

A pronounced negative impact on the SARS-CoV-2 immunization process was provided by the lack of information about COVID-19 vaccines (r = −0.638) and the unreliability of these data (r = −0.693), which determine the doubt about the vaccination effectiveness and safety (r = 0.674), and the fear of side effects and post-vaccination complications (r = 0.686). It was found that the percentage of students who decided to be vaccinated against SARS-CoV-2 was positively correlated with the percentage of students whose main information sources were medical professionals (r = 0.484), medical university (r = 0.584), and scientific conferences and manuscripts (r = 0.317). Information from the Internet (r = 0.164) and from familiar (r = −0.096) had no significant effect on student SARS-CoV-2 immunization attitudes. Students who received information mainly from the mass media vaccinated in a significantly lower percentage (r = −0.623). It should be emphasized that the percentage of young people who were negative about SARS-CoV-2 immunization (r = −0.450) and not fully decided on this issue (r = −0.722) was negatively correlated with the percentage of COVID-19-vaccinated students.

It was found that the student COVID-19 vaccination coverage did not depend on their student residence, i.e., whether they lived in a family (r = 0.055) or in a university dormitory (r = −0.043). A negative correlation was noted between the percentage of COVID-19-vaccinated students and the proportion of students who exclusively prefer foreign vaccines (r = −0.322). The percentage of future specialists who recommended COVID-19 vaccination to everyone around them and who, accordingly, largely affect the formation of collective population immunity, was significantly correlated with the percentage of students who were positive about SARS-CoV-2 immunization and had been vaccinated themselves (r = 0.903).

Study design and research results are summed up in flow chart diagram (Figure S1—Supplementary Materials).

## 4. Discussion

According to recent studies, the understanding of the importance of the COVID-19 vaccine among medical students all over the world is very high, since COVID is a potentially severe acute respiratory infection [28,36,37,38,39]. However, the presence of words such as ‘hesitancy’, ‘barriers’, and ‘refusal’ in the literature regarding COVID-19 vaccination demonstrates the clear problems of COVID-19 vaccine approval among the young medical community. It was shown that almost all Russian medical students believed that COVID-19 was a serious disease (89.3%) and expressed profound concern about the current epidemiological situation (89.9%), sounding similar to the common understanding of the situation among medical students from around the world; vaccination importance was recognized by most of the respondents in the known studies, reaching 73.2–99.4% [36,37,38,39].

Hesitancy in accepting COVID-19 vaccination has a place among medical students in different countries and in some studies its prevalence even reaches 23.0–46.0% [27,28,40]. In Russia, most future specialists (65.0%) consider active population immunization to be the most effective method of preventing infectious diseases and post-infectious complications, but only a third of them (39.0%) have a positive attitude towards COVID-19 vaccination, and 42.7% of respondents are still undecided on this issue, and show low intention to recommend COVID-19 vaccination to others (demonstration of ‘hesitancy’). This criterion does not exactly exceed the percentage reported in other studies, but it is very close to the highest negative level. The need to obtain COVID-19 vaccination certificates motivated students to get vaccinated more than the desire to prevent COVID-19 and its complications.

Therefore, the real COVID-19 vaccination rate among Russian medical students in autumn 2021 was shown to be 58.8 ± 7.69%, which did not differ from the COVID-19 vaccination coverage among the general population at that time, and was significantly lower than the target value for collective immunity, which, according to actual data of the Russian Federal Service on Customers’ Rights Protection and Human Well-Being Surveillance was 80%. The number of COVID-19-vaccinated medical students worldwide was also not sufficient and according to systematic analysis of 40 original articles published between January 2020 and December 2021 averaged 61.9% [41].

It was found that the main reason for COVID-19 vaccination hesitancy among medical students and the low vaccination rate in Russia is the lack of reliable information on COVID-19 vaccines, their composition, action mechanisms and contraindications, which determine the doubt about the effectiveness and safety of COVID-19 vaccination, and the fear of side effects and post-vaccination complications, which has also been emphasized by other scientists [28,33,34]. It has been proved that the percentage of students who decided to be vaccinated against SARS-CoV-2 was positively correlated with the percentage of students whose main sources of information were medical professionals, medical universities, scientific conferences, and manuscripts, which at that point provided the least information about active SARS-CoV-2 immunization to medical students. Students who received data mainly from the media were vaccinated in a significantly lower percentage because of anti-vaccination propaganda conducted there. Similar assumptions were made in other studies [28,36]. Lack of information about COVID-19 vaccines can be a source of stress for young medical specialists [42].

For the first time, it was found that the student COVID-19 vaccination rate was largely determined by the actual epidemiological situation of the region, and primarily by indicators such as mortality (r = 0.655) and lethality (r = 0.701), which may be related to medical students’ awareness of COVID-19 treatment options and care quality in corresponding regions. It was proved that student COVID-19 vaccination coverage rates were independent of gender and student residence. For the first time, our study demonstrated that the group of senior students had a higher number of COVID-19 vaccine completers than the group of junior students, which may be due to their greater involvement in coronavirus hospitals and more frequent contact with health care workers. Other authors also established that willingness to be vaccinated against COVID-19 among medical students generally increased with age and education [27].

It has been shown that the percentage of students willing to recommend COVID-19 vaccination to the people around them and thereby contribute to increasing the collective immunity level is significantly dependent on the percentage of students who have been COVID-19 vaccinated. Many scientists consider that medical students are among the front-line medical professionals who meet patients. In addition, it is very important to achieve high rates of COVID-19 vaccination coverage in this group of health care providers, as they will recommend COVID-19 vaccination and counsel vaccine-hesitant people [27]. Public attitudes toward COVID-19 vaccine prophylaxis are formed in communication with medical students and directly depend on their competence in this matter. The ability to competently convey to the patient the essence of SARS-CoV-2 immunologic prophylaxis, to justify its necessity, to explain the existing questions in an accessible language, to inform about possible adverse reactions are the most important student skills, which they should receive, first, at a medical university.

One of our study limitations was the lack of result separation by faculty, as there is a perception that medical student knowledge varies between different specializations. We consider all respondents to have a high level of education corresponding to their course. In addition, the relatively small number of males compared to females may likely be a limiting factor on the study results. In future studies we will choose proportional numbers of students from all medical universities. Another limitation is that the survey was conducted under the condition that only Russian vaccines were included. For this reason, this study and its results must be understood as the initial stage of multi-central research for COVID-19 vaccination in order to understand its problems.

## 5. Conclusions

The real COVID-19 vaccination rate among Russian medical students in autumn 2021 was 58.8 ± 7.69%, which did not differ from the COVID-19 vaccination coverage of the general population at that time, and was significantly lower than the target value for collective immunity. The main reason for low COVID-19 vaccination among medical students’ rate is the lack of reliable information about COVID-19 vaccines. Significant information sources influencing students’ attitudes toward vaccination include medical professionals, medical universities, academic conferences, and manuscripts. The group of senior students had a higher number of COVID-19 vaccine completers than the group of junior students.

The obtained results support the following recommendations:

(1) The primary task is to eliminate the information deficiency about COVID-19 vaccines among medical students using educational resources and, above all, the medical university, considering its significance in the forming adherence to SARS-CoV-2 immunologic prophylaxis. (2) SARS-CoV-2 immunologic prophylaxis education should be organized to all students, focusing on junior students. (3) Registration of foreign COVID-19 vaccines in Russia and development of official recommendations for their use will increase the percentage of vaccinated students and population. (4) A conscious decision by medical students to be vaccinated against SARS-CoV-2 will contribute to their competent explanation of immunologic prophylaxis essence to the population and increase collective immunity.

## Figures and Tables

**Table 1:** Number and percentage of surveyed medical students.

Russian Higher Education Institution	Number and Percentage of Respondents	Total Number of Students
I.M. Sechenov First Moscow State Medical University (Sechenov University)	575 (13.2%)	4356
Mechnikov North-West State Medical University	302 (10.8%)	2797
Saratov State Medical University named after V.I. Razumovsky (Razumovsky University)	283 (12.9%)	2194
Smolensk State Medical University	215 (12.6%)	1707
Northern State Medical University	290 (11.7%)	2479
Federal State Autonomous Educational Institution of Higher Education “M. K. Ammosov North-Eastern Federal University”	212 (11.5%)	1845
Kirov State Medical University	344 (13.6%)	2529
Kazan State Medical University	469 (15.1%)	3106
Penza State Medical University	200 (11.9%)	1681
Total	2890 (12.7%)	22,694

**Table 2:** Mean age of surveyed medical students and their number according to sex and grade.

Russian Higher Education Institution	Mean Age	1–3 Year	4–6 Year	Males	Females
I.M. Sechenov First Moscow State Medical University (Sechenov University)	19.53 ± 3.01	299	276	133	442
Mechnikov North-West State Medical University	19.96 ± 2.32	162	140	75	227
Saratov State Medical University named after V.I. Razumovsky (Razumovsky University)	19.65 ± 2.64	157	126	80	203
Smolensk State Medical University	20.81 ± 2.13	84	131	67	148
Northern State Medical University	21.49 ± 1.96	127	163	62	228
Federal State Autonomous Educational Institution of Higher Education “M. K. Ammosov North-Eastern Federal University”	22.88 ± 3.67	108	104	84	128
Kirov State Medical University	22.05 ± 2.56	170	174	88	256
Kazan State Medical University	20.47 ± 2.46	277	192	141	328
Penza State Medical University	22.10 ± 1.79	83	117	70	130
Total	20.99 ± 2.28	1467 (50.8%)	1423 (49.2%)	800 (27.7%)	2090 (72.3%)

## Data Availability

The data are not publicly available due to Local Ethical Committee requirements.

## Supplementary Materials

The following supporting information can be downloaded at: https://www.mdpi.com/article/10.3390/ijerph191811556/s1, Figure S1. Flow research chart.
