# Supplementary figures and images for: COVID-19 Vaccinating Russian Medical Students—Challenges and Solutions: A Cross-Sectional Study
Source: Int J Environ Res Public Health. 2022 Sep 14;19(18):11556. doi: 10.3390/ijerph191811556 (PMC9517622; doi:10.3390/ijerph191811556)

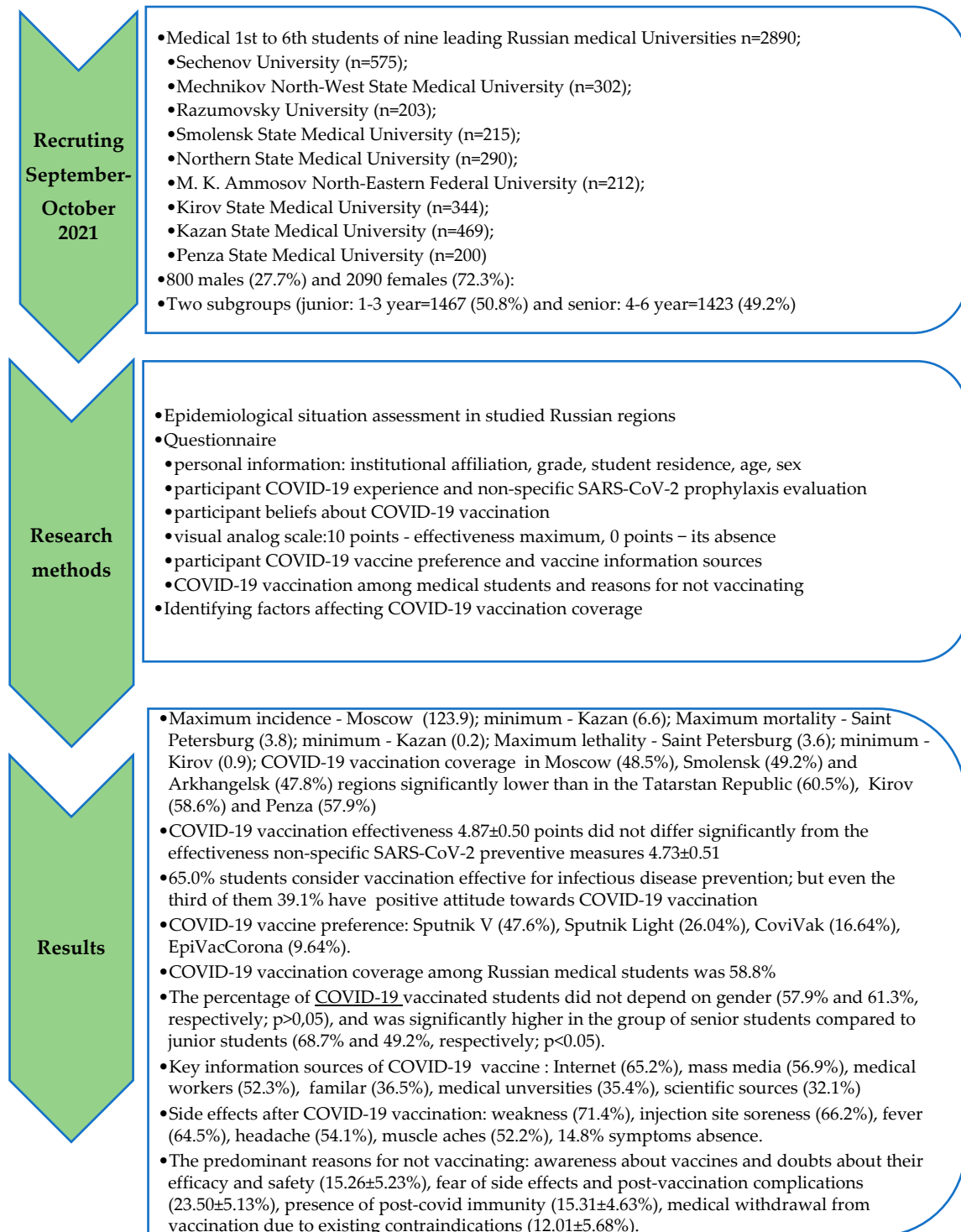

**Figure S1.** Flow research chart.

Supplement: Supplementary file 1 [file ijerph-19-11556-s001.zip › ijerph-1855598-supplementary.pdf]
